# Supplementary material for: Combining interventions to reduce the spread of viral misinformation
Source: Nat Hum Behav. 2022 Jun 23;6(10):1372–80. doi: 10.1038/s41562-022-01388-6 (PMC9584817; doi:10.1038/s41562-022-01388-6)
Supplement: Supplementary file 1 — Supplementary figures and tables. [file 41562_2022_1388_MOESM1_ESM.pdf]

nature portfolio

https://doi.org/10.1038/s41562-022-01388-6

Supplementary information

Combining interventions to reduce the spread of viral misinformation

In the format provided by the authors and unedited

Supplementary Information: Combining interventions to reduce the spread of viral misinformation

Joseph B. Bak-Coleman[1,2,3*], Ian Kennedy[1,4], Morgan Wack[1,5], Andrew Beers[1,6], Joseph S Schafer[1,6], Emma S. Spiro[1,3,4], Kate Starbird[1,7] and Jevin D. West[1,3]

[1]Center for an Informed Public, University of Washington, Seattle, WA 98195.
[2]eScience Institute, University of Washington, Seattle, WA 98195.
[3]The Information School, University of Washington, Seattle, WA 98195.
[4]Department of Sociology, University of Washington, Seattle, WA 98195.
[5]Department of Political Science, University of Washington, Seattle, WA 98195.
[6]Paul G. Allen School of Computer Science and Engineering, University of Washington, Seattle, WA 98195.
[7]Human Centered Design and Engineering, University of Washington, Seattle, WA 98195.

*Corresponding author(s). E-mail(s): jbakcoleman@gmail.com;

Supplementary Figures

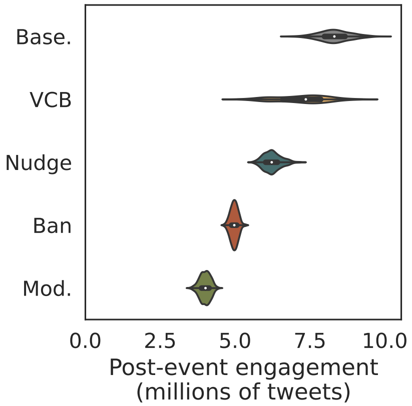

Supplementary Figure 1 Estimated engagement following the largest event for modest control policies. V viral circuit breakers are employed for 5% of content, reducing virality by 10% after 2 hours. Among the content subjected to a viral circuit breaker, 20% is subsequently removed after four hours. This is done alongside a 10% nudge. Finally, accounts that have been removed remain banned, and a 3-strikes policy is applied to verified accounts and those with more than 100 thousand followers.Violins indicate the simulated distribution of total posts across all events. Dots and lines within violins indicate median and innerquartile range.

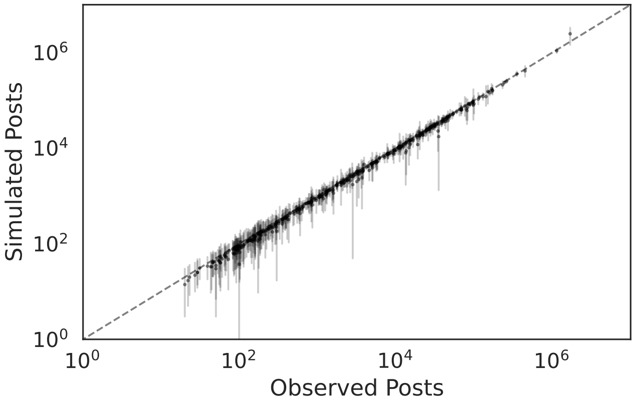

Supplementary Figure 2 Figure capturing cumulative simulated posts (x axis) vs observed posts (y axis). Points indicate the simulated median, and lines indicate the region wherein 89% of simulations fall.

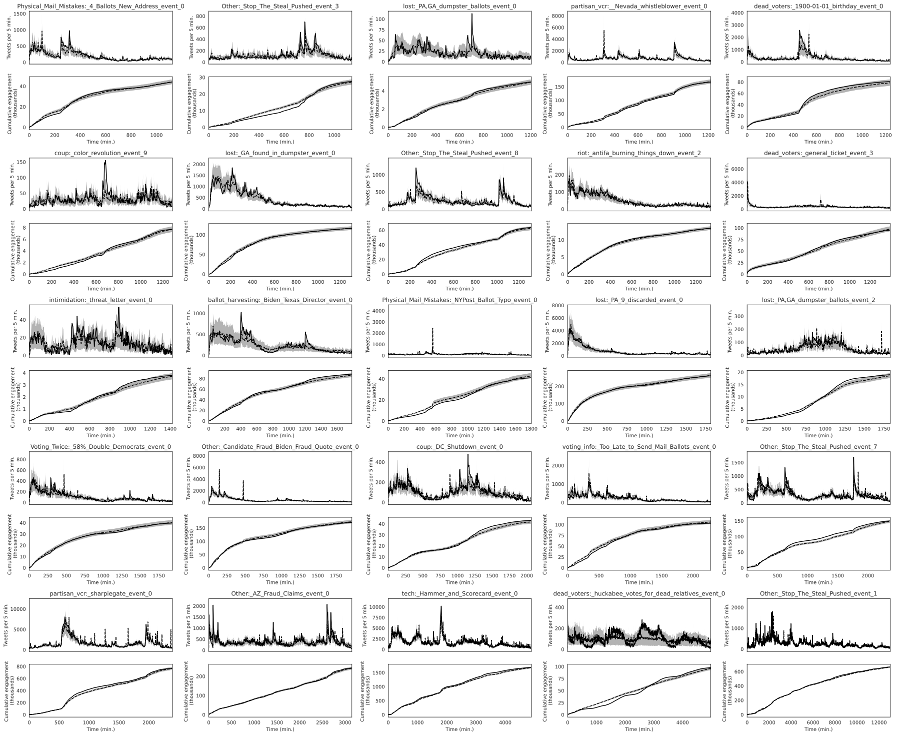

Supplementary Figure 3 Posterior predictive plots of time-series for the largest 25 events captured. For each pair of plots, the top indicates the raw time-series and the lower plot indicates cumulative posts. Shaded grey regions are 89% credible intervals, dashed-lines indicate the posterior-predictive mean, and solid lines indicate the observed data.

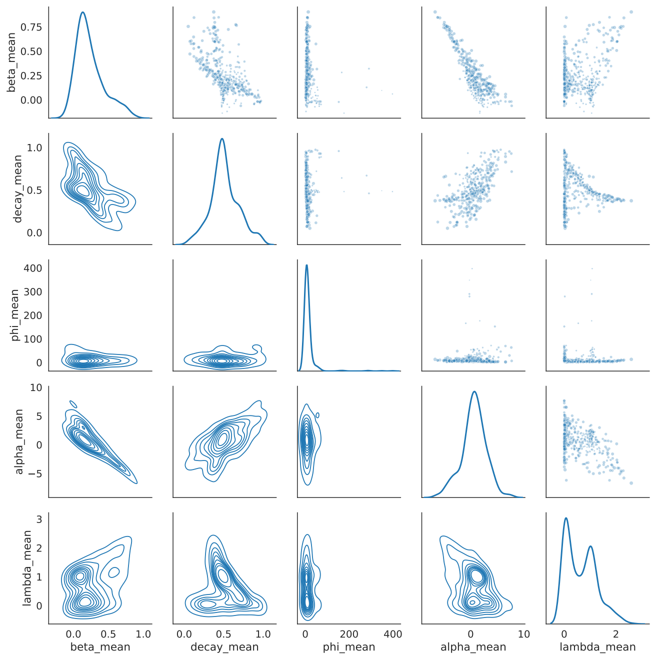

Supplementary Figure 4 Pairplot showcasing the pair-wise joint posterior distribution for model parameters across all included events.

Delay (minutes)	μ (% decrease)	5.5%	94.5%
baseline	10815700 (-0.0)	9795039 (-0.0)	11663709 (-0.0)
240	4805308 (-55.6)	4571121 (-59.2)	5003092 (-50.7)
120	3095625 (-71.3)	2953594 (-73.8)	3235433 (-68.2)
60	1591997 (-85.2)	1505854 (-86.6)	1691129 (-83.4)
30	669553 (-93.8)	622658 (-94.4)	725078 (-92.9)
15	225007 (-97.9)	203949 (-98.2)	246314 (-97.6)

Supplementary Table 1 Table of effects for time-lagged removal of all misinformation events. Listed are the median outcome (μ) and the 89% credible intervals. Figures are computed as the sum across all included events and 500 simulations for each event.

Delay (minutes)	μ (% decrease)	5.5%	94.5%
baseline	10815700 (-0.0)	9795039 (-0.0)	11663709 (-0.0)
240	9858597 (-9.4)	7721522 (-29.6)	10986300 (5.5)
120	9648110 (-11.1)	7135560 (-34.7)	10706212 (4.4)
60	9181366 (-15.6)	6613025 (-37.9)	10449720 (0.2)
30	9076799 (-16.5)	6515272 (-41.4)	10217865 (-1.4)
15	8961931 (-17.4)	6505631 (-40.1)	10255932 (-2.3)

Supplementary Table 2 Table of effects for time-lagged removal of 20% of misinformation events. Listed are the median outcome (μ) and the 89% credible intervals. Figures are computed as the sum across all included events and 500 simulations for each event.

Delay (minutes)	μ (% decrease)	5.5%	94.5%
baseline	10815700 (-0.0)	9795039 (-0.0)	11663709 (-0.0)
240	5890581 (-45.3)	5699910 (-49.8)	6087193 (-39.4)
120	4839277 (-55.2)	4707706 (-58.9)	4998551 (-50.1)
60	3883683 (-63.9)	3767271 (-66.9)	4025288 (-60.2)
30	3357979 (-68.9)	3245505 (-71.5)	3456819 (-65.4)
15	3098563 (-71.3)	2999018 (-73.7)	3193319 (-68.3)

Supplementary Table 3 Table of effects for a time-lagged virality circuit breaker that reduces virality for all events by 10%. Listed are the median outcome (μ) and the 89% credible intervals. Figures are computed as the sum across all included events and 500 simulations for each event.

Delay (minutes)	μ (% decrease)	5.5%	94.5%
baseline	10815700 (-0.0)	9795039 (-0.0)	11663709 (-0.0)
240	10062860 (-7.5)	7957802 (-27.5)	11159151 (6.7)
120	9837051 (-9.7)	7690466 (-30.2)	10793311 (5.5)
60	9667358 (-11.8)	7088186 (-34.6)	10870867 (4.9)
30	9637591 (-11.7)	6999906 (-35.6)	10781698 (4.5)
15	9579818 (-11.8)	7135440 (-35.8)	10658453 (2.8)

Supplementary Table 4 Table of effects for a time-lagged virality circuit breaker that reduces virality for 20% events by 10%. Listed are the median outcome (μ) and the 89% credible intervals. Figures are computed as the sum across all included events and 500 simulations for each event.

Nudge	μ (% decrease)	5.5%	94.5%
baseline	10815700 (-0.0)	9795039 (-0.0)	11663709 (-0.0)
5%	9223434 (-15.2)	8366277 (-24.3)	9935823 (-3.0)
10%	7886847 (-26.4)	7451354 (-33.5)	8533658 (-18.1)
20%	6430541 (-40.3)	6240892 (-45.1)	6646983 (-34.0)
40%	4786377 (-55.6)	4653868 (-59.0)	4918860 (-50.9)

Supplementary Table 5 Table of effects for nudges that reduce the proportion of users susceptible or exposed to information by a given percent. Listed are the median outcome (μ) and the 89% credible intervals. Figures are computed as the sum across all included events and 500 simulations for each event.

Policy	μ (% decrease)	5.5%	94.5%
baseline	10815700 (-0.0)	9795039 (-0.0)	11663709 (-0.0)
verified	9410894 (-12.7)	8567313 (-23.0)	10093597 (-2.3)
currently removed	7502763 (-30.6)	7247613 (-36.1)	7813552 (-22.3)

Supplementary Table 6 Table of effects for the cumulative amount of misinformation at baseline, with the removal of currently banned individuals, and after implementation of a 3-strikes policy applied to verified users. Listed are the median outcome (μ) and the 89% credible intervals. Figures are computed as the sum across all included events and 500 simulations for each event.

Policy	μ (% decrease)	5.5%	94.5%
baseline	10815700 (-0.0)	9795039 (-0.0)	11663709 (-0.0)
1×10^4	4353111 (-59.7)	4249263 (-63.0)	4466539 (-55.0)
5×10^4	6200849 (-42.6)	6053426 (-46.8)	6379307 (-36.1)
1×10^5	7464753 (-31.0)	7053591 (-37.1)	7896123 (-22.7)
5×10^5	9869939 (-8.7)	9008353 (-18.4)	10575005 (3.2)

Supplementary Table 7 Table of effects for the cumulative amount of misinformation at baseline and after implementation of a 3-strikes policy applied to accounts with more than a given number of followers. Listed are the median outcome (μ) and the 89% credible intervals. Figures are computed as the sum across all included events and 500 simulations for each event.

Policy	μ (% decrease)	5.5%	94.5%
Baseline	10815700 (-0.0)	9795039 (-0.0)	11663709 (-0.0)
Decay	9541255 (-11.5)	7415450 (-31.7)	10881188 (3.2)
Nudge	7882401 (-26.5)	7435311 (-33.7)	8529590 (-17.1)
Ban	6241553 (-42.1)	6063285 (-46.9)	6455361 (-35.6)
Modest	5027687 (-53.5)	4667963 (-58.2)	5300793 (-48.2)

Supplementary Table 8 Table of effects for interventions applied to modest intervention either individually or combined. These effects assume a 10% virality circuit breaker enacted at 120 minutes, with 20% of events impacted by the virality circuit breaker removed at 240 minutes. It further assumes that those currently banned remain banned and a three strikes policy is applied to users with more than 100K followers, and a 10% reduction in individual sharing or exposure to misinformation is achieved via a nudge or similar policy. Listed are the median outcome (μ) and the 89% credible intervals. Figures are computed as the sum across all included events and 500 simulations for each event.

Springer Nature 2021 LaTeX template

Policy	μ (% decrease)	5.5%	94.5%
Baseline	10815700 (-0.0)	9795039 (-0.0)	11663709 (-0.0)
Decay	8885435 (-18.7)	6392408 (-41.2)	10277717 (-1.3)
Nudge	6432815 (-40.4)	6250430 (-45.0)	6659200 (-34.0)
Ban	5779744 (-46.3)	5642697 (-50.5)	5938714 (-40.9)
Aggressive	3973074 (-63.0)	3695881 (-66.9)	4258272 (-58.4)

Supplementary Table 9 Table of effects for interventions applied to more aggressive intervention either individually or combined. These effects assume a 20% virality circuit breaker enacted at 60 minutes, with 20% of events impacted by the virality circuit breaker removed at 120 minutes. It further assumes that those currently banned remain banned and a three strikes policy is applied to users with more than 50 thousand followers, with a 20% reduction in individual sharing or exposure to misinformation is achieved via a nudge or similar policy.Listed are the median outcome (μ) and the 89% credible intervals. Figures are computed as the sum across all included events and 500 simulations for each event.

Parameter (minutes)	μ (% decrease)	5.5%	94.5%
β	0.94	0.92	0.95
σ	1.30	1.2	1.4

Supplementary Table 10 Table of effects for Bayesian log-normal regression predicting subsequent engagement as a function of the logarithm of within-event engagement.
