# Supplementary material for: Combining interventions to reduce the spread of viral misinformation
Source: Nat Hum Behav. 2022 Jun 23;6(10):1372–80. doi: 10.1038/s41562-022-01388-6 (PMC9584817; doi:10.1038/s41562-022-01388-6)
Supplement: Supplementary file 2 — Reporting Summary [file 41562_2022_1388_MOESM2_ESM.pdf]

nature portfolio

Corresponding author(s): Joseph Bak-Coleman

Last updated by author(s): May 26, 2022

Reporting Summary

Nature Portfolio wishes to improve the reproducibility of the work that we publish. This form provides structure for consistency and transparency in reporting. For further information on Nature Portfolio policies, see our Editorial Policies and the Editorial Policy Checklist.

Statistics

For all statistical analyses, confirm that the following items are present in the figure legend, table legend, main text, or Methods section.

n/a	Confirmed	
☒	☐	The exact sample size (*n*) for each experimental group/condition, given as a discrete number and unit of measurement
☒	☐	A statement on whether measurements were taken from distinct samples or whether the same sample was measured repeatedly
☒	☐	The statistical test(s) used AND whether they are one- or two-sided<br>*Only common tests should be described solely by name; describe more complex techniques in the Methods section.*
☐	☒	A description of all covariates tested
☐	☒	A description of any assumptions or corrections, such as tests of normality and adjustment for multiple comparisons
☐	☒	A full description of the statistical parameters including central tendency (e.g. means) or other basic estimates (e.g. regression coefficient) AND variation (e.g. standard deviation) or associated estimates of uncertainty (e.g. confidence intervals)
☒	☐	For null hypothesis testing, the test statistic (e.g. *F*, *t*, *r*) with confidence intervals, effect sizes, degrees of freedom and *P* value noted<br>*Give P values as exact values whenever suitable.*
☐	☒	For Bayesian analysis, information on the choice of priors and Markov chain Monte Carlo settings
☐	☒	For hierarchical and complex designs, identification of the appropriate level for tests and full reporting of outcomes
☐	☒	Estimates of effect sizes (e.g. Cohen's *d*, Pearson's *r*), indicating how they were calculated

Our web collection on statistics for biologists contains articles on many of the points above.

Software and code

Policy information about availability of computer code

Data collection	Data were collected using the Twitter API in real time during the 2020 US presidential Election. Collection began on 1 September 2020 and ran through Dec 15th, 2020. Collection involved an evolving set of keywords in response to emerging narratives regarding electoral misinformation identified by the Electoral Integrity Partnership.
Data analysis	All data analysis was conducting in Python (Python 3.9.10) leveraging the standard Scipy stack and Pystan (v. 2.19.1.1). Versions of all installed python packages are available in the "requirements.txt" file of the code respository. Code to reproduce the results is available on Zenodo (https://doi.org/10.5281/zenodo.6478446). Any updates to the code can be found on Github (https://github.com/josephbb/CombinedPoliciesMisinfo)

For manuscripts utilizing custom algorithms or software that are central to the research but not yet described in published literature, software must be made available to editors and reviewers. We strongly encourage code deposition in a community repository (e.g. GitHub). See the Nature Portfolio guidelines for submitting code & software for further information.

Data

Policy information about availability of data

All manuscripts must include a data availability statement. This statement should provide the following information, where applicable:

- Accession codes, unique identifiers, or web links for publicly available datasets
- A description of any restrictions on data availability
- For clinical datasets or third party data, please ensure that the statement adheres to our policy

Given Twitter's Data Use-Agreement, we cannot release the full dataset. However, we have made available aggregated time-series sufficient to reproduce our findings. Data to reproduce the results are available on Zenodo (https://doi.org/10.5281/zenodo.6480218).

Field-specific reporting

Please select the one below that is the best fit for your research. If you are not sure, read the appropriate sections before making your selection.

☐ Life sciences ☒ Behavioural & social sciences ☐ Ecological, evolutionary & environmental sciences

For a reference copy of the document with all sections, see nature.com/documents/nr-reporting-summary-flat.pdf

Behavioural & social sciences study design

All studies must disclose on these points even when the disclosure is negative.

Study description	Our study involved deriving a model of viral information spread to examine the impact of interventions aimed at reducing the spread of viral misinformation. To do so, we collected a large number of Tweets related to the US 2020 Presidential election. Mixed-methods approaches were used to identify distinct incidents (specific narratives) of misinformation and associated search terms. These search terms were used to distinguish incidents and extract relevant tweets from our larger database. We then used quantitative methods to segment incidents into distinct events. Finally, we used this dataset to derive a mathematical model of misinformation spread and examine the likely impact of commonly proposed interventions.
Research sample	Our sample involves users on Twitter that posted about topics related to the 2020 US presidential elections. Given the large initial volume of Tweets collected and the large team of individuals used to identify and collect misinformation narratives, we believe the dataset is representative of English-language tweets in the US about the 2020 presidential election. This sample was used as the electoral integrity partnership and Twitter's open API provided a unique opportunity to gather the data needed to apply our approach.
Sampling strategy	We sampled randomly and used all tweets that matched the criteria. A sample size calculation was not necessary.
Data collection	All data were collected in accordance with the University of Washington Institutional Review Board. Our dataset was collected in real-time during the 2020 US election. We relied on a set of 160 keywords to collect posts from Twitter's API (1.04 billion). Keywords were updated in response to new narratives, for instance, adding "sharpiegate" and related terms after false narratives emerged about the use of Sharpie markers invalidating ballots. Working with the Electoral Integrity Partnership, we cataloged instances of false or misleading narratives that were either detected by the team or reported by external partners.. This led to a large corpus of tickets associated with validated reports of misleading, viral information about election integrity.<br><br>Tickets that shared a common theme were consolidated into incidents. We developed search terms and a relevant date range for each incident to query posts from our tweet database. Incidents (N=430) were generally characterized by one or more periods of intense activity followed by returning to a baseline state. Search terms and descriptions of incidents are provided along with the data.
Timing	September 1st 2020 to December 15th 2020
Data exclusions	Our model was fit to each event using PyStan 2.9.1.1. We fit events separately (rather than hierarchically) as they varied widely in their time scales, magnitudes, and context within the broader 2020 election cycle. Of the 544 candidate events, our model performed well on 454 events (10.4 Million posts/tweets) of rapid misinformation spread. Our model was unlikely to be suitable for all events as it makes the assumption that post volume is well predicted by the number of previously exposed accounts on Twitter.<br><br>To safeguard against this, we relied on a number of criteria to ensure model fit to a given event. Events were included in the final analysis if a) the posterior 89% C.I. of total posts contained the observed value and b) the chains successfully converged for all parameters (r_hat < 1.1) c) The fit did not contain divergent transitions and d) the event lasted longer than an hour (i.e., >12 data points to fit). Other than these criteria, events surrounding the Dominion narrative were removed as they involved long periods high volume online discussion. This filtering process resulted in the inclusion of 454 events (83% of total events), and 10.4 million posts.
Non-participation	Analyses were conducted on digital trace data, and no participants were explicitly used.

Randomization	This study does not have explicit experimental and control groups, so no randomization was needed.

Reporting for specific materials, systems and methods

We require information from authors about some types of materials, experimental systems and methods used in many studies. Here, indicate whether each material, system or method listed is relevant to your study. If you are not sure if a list item applies to your research, read the appropriate section before selecting a response.

Materials & experimental systems

n/a	Involved in the study	
☒	☐	Antibodies
☒	☐	Eukaryotic cell lines
☒	☐	Palaeontology and archaeology
☒	☐	Animals and other organisms
☐	☒	Human research participants
☒	☐	Clinical data
☒	☐	Dual use research of concern

Methods

n/a	Involved in the study	
☒	☐	ChIP-seq
☒	☐	Flow cytometry
☒	☐	MRI-based neuroimaging

Human research participants

Policy information about studies involving human research participants

Population characteristics	Users on Twitter
Recruitment	Data were collected via the API as they were publicly available.
Ethics oversight	University of Washington IRB

Note that full information on the approval of the study protocol must also be provided in the manuscript.
